# Changing Incidence and Stage Distribution of Prostate Cancer in a Lithuanian Population—Evidence from National PSA-Based Screening Program

**DOI:** 10.3390/ijerph16234856

**Published:** 2019-12-03

**Authors:** Ausvydas Patasius, Giedre Smailyte

**Affiliations:** 1 Laboratory of Cancer Epidemiology, National Cancer Institute, LT-08406 Vilnius, Lithuania; giedre.smailyte@nvi.lt; 2 Institute of Health Sciences, Faculty of Medicine, Vilnius University, LT-03101 Vilnius, Lithuania

**Keywords:** incidence, prostate cancer, PSA-based screening, stage migration

## Abstract

Background: The aim of this study was to examine the impact of screening introduction on prostate cancer incidence changes, and changes in stage distribution in Lithuania between 1998–2016. Methods: Age-standardized incidence as well as stage-specific incidence rates were calculated. Joinpoint regression was used to estimate the annual percentage change in the incidence changes by determined stage: Localized, advanced, distant and unknown. Results: Over the study period, a total number of 48,815 new prostate cancer cases was identified. Age-standardized incidence rose from 51.9 per 100,000 in 1998 to 279.3 per 100,000 in 2007 (by 20.3% per year) and then decreased thereafter by 3.8% annually. Highest incidence rates after introduction of prostate specific antigene (PSA)-based screening was found for localized disease, followed by advanced. Incidence of localized disease rose by 38.2% per year until 2007 reaching the highest rate of 284.6 per 100,000, with a subsequent decrease of 5.5% every year thereafter. Advanced stage of disease experienced rise till 2007, and continuous decrease by 11.1% every year thereafter. Incidence of disease with distant metastasis was lowest, and rose till 2003, thereafter incidence significantly decreased by 8.1% every year. Conclusions: To our knowledge, this is the first report of stage migration effect in Lithuania, following the introduction of nationwide PSA-based screening. Prostate cancer screening substantially increased the overall incidence and incidence of localized cancer.

## 1. Introduction

Prostate cancer was estimated to be the third most common cancer in Europe in 2018, with 450,000 cases, as well as the fifth leading cause of cancer death, with 107,000 deaths [1]. Widespread implementation of prostate specific antigen (PSA) testing has changed the epidemiologic situation of prostate cancer worldwide. There has been a noted incidence rise in prostate cancer in most European countries and worldwide [2,3]. Despite recommendation from international health authorities [4], in 2006, Lithuania started the Early Prostate Cancer Detection Program (EPCDP). Lithuania is the only country in the world with a running nation-wide PSA-based prostate cancer screening program. Asymptomatic men with PSA levels >3 ng/mL are offered further urological evaluation, including digital rectal examination and transrectal ultrasound. The decision to perform a prostate biopsy is at the discretion of the consulting urologist. The EPCDP experienced several modifications for check-up frequency. At first, screening annually targeted men aged 50–75 years and younger men (>45 years) with a family history of prostate cancer. From 2009 to 2016, screening was performed every two years By the end of 2010, 72 to 78% of the total eligible male population received at least one PSA test [5]. Costs of EPCDP varied between 1.26 million euros in 2006 to 2.1 million euros in 2008 [6]. Changes of prostate cancer incidence and mortality have been analyzed and interpreted: Lithuania experienced continuous increase of prostate cancer incidence steadily, from 37.03 per 100,000 in 1994, to 75.66 per 100,000 in 2001. During the period between 2001–2007, there was a rapid annual increase of 23% in prostate cancer incidence. A peak incidence rate of 279.33 per 100,000 (European standard) was reached in 2007, after the start of the screening program. Thereafter, these incidence changes led to a decrease in the incidence rate. Mortality in Lithuania had been continuously growing, however, in 2006 mortality started to decrease by 1.4% annually [7]. This decrease is unlikely attributable to the success of the early effects of EPCDP, as the start of decrease is the same as the start of the program. However, analyzed changes are lacking detailed information about disease distribution by stage. The aim of this study was to examine the impact of screening introduction on prostate cancer incidence changes, and changes in stage distribution in Lithuania between 1998–2016.

## 2. Materials and Methods

The study is based on all new prostate cancer cases identified in the Lithuanian Cancer Registry during 1998–2016. The Lithuanian Cancer Registry is a population-based registry which contains personal and demographic information (place of residence, sex, date of birth, vital status), as well as information on diagnosis (cancer site, date of diagnosis, stage of disease, method of cancer verification) and death (date of death, cause of death) of all cancer patients in Lithuania. Age-standardized incidence, as well as group-specific incidence rates were calculated using the direct method (Europe standard population) [8]. Corresponding population data by age and year were available from Statistics Lithuania. We determined three prostate cancer groups by cancer stage and TNM (Tumor, Node, Metastasis): Localized (T1-T2N0M0 or stage I–II), advanced (T3-T4N0M0 or stage III), distant (any T, N1 or M1, or stage IV) and unknown. Changes in overall incidence, and incidence changes by stage of disease were examined.

The joinpoint regression model was used to provide estimated annual percentage change (APC), and to detect points in time where significant changes in the trends occur. For each of the identified trends, we also fitted a regression line to the natural logarithm of the rates, using calendar year as a regression variable. Joinpoint regression analysis is often used when the temporal trend of a given quantity—like incidence, prevalence and mortality—is the field of interest. Permutation tests for joinpoint regression analysis were introduced and proposed to be applied to cancer rates by Kim et al. in the 2000s [9]. A maximum number of one joinpoint was decided a priori, in order to identify the most important time point at which stage incidence changes occurred. Using 95% confidence intervals, APC was calculated. Changes were considered statistically significant if *p* < 0.05. A Monte Carlo permutation method was used for the tests of significance [9]. Joinpoint analysis was performed for all age groups and for each outspread group of prostate cancer. Joinpoint software version 4.3.1.0 (Information Management Services, Inc., Calverton, MD, USA) was used.

## 3. Results

**Figure 1:**
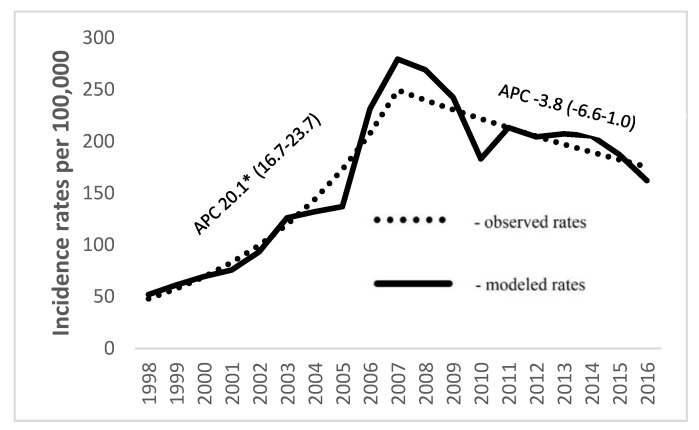
Age-adjusted prostate cancer incidence rates in Lithuania from 1998 to 2016 (* statistically significant; APC: Annual percentage change).

**Figure 2:**
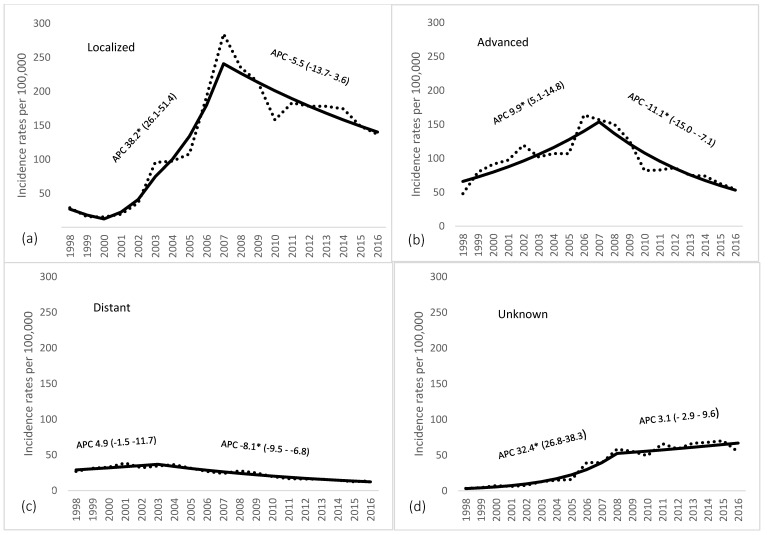
Changes in incidence rates of prostate cancer by stage in Lithuania 1998–2016; ((**a**): Localized; (**b**): Advanced; (**c**): Distant; (**d**): Unknown) (* statistically significant; APC: Annual percentage change).

Over the study period, a total number of 48,815 new prostate cancer cases was identified. Description of study population is presented in Table 1. Age-standardized incidence rose from 51.9 per 100,000 in 1998 to 279.3 per 100,000 in 2007 (by 20.3% per year), and then decreased thereafter by 3.8% annually (Figure 1). Figure 2 shows trends of prostate cancer incidence by stage of disease. Highest incidence rates after introduction of PSA-based screening were found for localized disease, followed by advanced. Incidence of localized disease rose by 38.2% per year until 2007, reaching the highest rate at 284.6 per 100,000, with a subsequent decrease by 5.5% every year. Advanced stage of disease experienced a rise until 2007, followed by a continuous decrease by 11.1% every year thereafter. Incidence of disease with distant metastasis was lowest and rose till 2003, and significantly decreased by 8.1% every year thereafter.

## 4. Discussion

The results of this analysis provide an overview of the incidence changes in stage distribution of prostate cancer in Lithuania over the past two decades, following the introduction of a national PSA-based screening program. One of the main endpoints of successful screening, and sign of the effectiveness of PSA in detecting significant new cancers is a reduction of mortality from prostate cancer [10,11,12]. The existing evidence from the European Randomized Study of Screening for Prostate Cancer has showed, that screening results led to a 21% prostate cancer mortality reduction in an intention-to-treat analysis [13]. However, mortality risk reduction in randomized prostate cancer screening trials remains weakly tangible [14]. Other signs of successful screening, that appear much earlier than mortality reduction can be seen, are increase of incidence of disease followed by decrease thereafter, and a downward shift in age and in the stage of disease at diagnosis [12]. Results of our study show an incidence decrease in advanced and distant forms of the disease, with an overall increase of localized disease after the introduction of PSA-based screening at the population level.

Incidence peak, seen after introduction of prostate cancer screening, was observed in the United States in the early 1990s [15]. This phenomena is called “backlog”, described by A. Farkas in 1998 and C.Mettlin in 2000. The wide use and utilization of PSA testing as a screening tool detects clinically insignificant cancers, and results in a largely increasing incidence for this disease. As soon as this backlog is diagnosed, incidence exhibits a downfall, reaching true level of incidence which is higher than in the pre-screening era [16,17]. Overall, prostate cancer incidence changes in Lithuania mimicked changes of incidence in the USA in the early 1990s [1]. There has been a rapid incidence peak since the start of screening program, followed by a decrease thereafter. In Lithuania, PSA became available in 2000, and in 2006, a nationwide PSA-based prostate cancer early detection program was started [7]. Since the start of the program, in the period between 2006–2010 around 72–78% of the total eligible male population received at least one PSA test [5].

Introduction of the EPCDP in Lithuania resulted in an incidence peak in 2007 for advanced and localized cancer stages. The incidence peak for distant stage of disease was seen in 2001, although incidence for systemic disease had continuously decreased from 2003 to 2016. Observed changes in incidence could be a result of an increase in transurethral resection of the prostate for obstructive symptoms, and later because of PSA introduction into clinical practices in 2000. Rapid decline in incidence of advanced stage disease shows early detection related effect before the start of the national EPCDP. Similar incidence changes by stage were observed in the United States and Tyrol (Europe), after introduction of population-based prostate cancer screening [18]. Analysis of stage distribution in Lithuania after implementation of PSA into clinical practice and introduction of EPCDP, revealed clear incidence reduction of advanced disease and stage with distant metastasis.

The main limitation of this study is the proportion of unknown stages of disease and/or staging by TNM classification. Based on the results of our recent study, patients with a lack of staging information are likely to live like patients in the advanced stage group. It suggests that patients with an unknown stage of disease are not presenting a metastatic status of the disease [5]. It is notable that incidence in the unknown stage group rose from the beginning of the study to 2008.

Not only can the improvement in age and stage distribution be attributable to the test itself, but also to an overall improvement in the knowledge and cancer awareness at the population level. Often screening programs are accompanied by the widespread publication of screening information. Studies from other cancer site screening programs, analyzing degree of caution in stage I disease, showed that the proportion of stage I diseases may well reduce with consecutive screening rounds [19].

## 5. Conclusions

To our knowledge, this is the first report of stage migration effect in Lithuania, following the introduction of the nationwide PSA-based screening. Prostate cancer screening substantially increased the overall incidence and incidence of localized cancer.

## Figures and Tables

**Table 1:** Study group characteristics.

		Number of Cases	%	Before Screening (1998–2005)	%	During Screening (2006–2016)	%
	**Overall**	48,815	100.00	11,400	100.00	37,415	100.00
**By stage**	Localized	22,095	45.26	3706	32.51	18,389	49.15
	Advanced	12,593	25.80	4910	43.07	7683	20.53
	Distant	3150	6.45	1688	14.81	1462	3.91
	Unknown	10,977	22.49	1096	9.61	9881	26.41
**By age group**	**<50 years**	509	100.00	67	100.00	442	100.00
	Localized	314	61.69	25	37.31	289	65.39
	Advanced	68	13.36	16	23.88	52	11.76
	Distant	38	7.47	19	28.36	19	4.30
	Unknown	89	17.49	7	10.45	82	18.55
	**50–74 years**	37,006	100.00	7204	100.00	29,802	100.00
	Localized	18,349	49.58	2582	35.84	15,767	52.91
	Advanced	8646	23.36	3028	42.03	5618	18.85
	Distant	1934	5.23	1045	14.51	889	2.98
	Unknown	8077	21.83	549	7.62	7528	25.26
	**>75 years**	11,300	100.00	4129	100.00	7171	100.00
	Localized	3432	30.37	1099	26.62	2333	32.53
	Advanced	3879	34.33	1866	45.19	2013	28.07
	Distant	1178	10.42	624	15.11	554	7.73
	Unknown	2811	24.88	540	13.08	2271	31.67

## References

[1] Ferlay J., Colombet M., Soerjomataram I., Dyba T., Randi G., Bettio M., Gavin A., Visser O., Bray F. (2018). Cancer incidence and mortality patterns in Europe: Estimates for 40 countries and 25 major cancers in 2018. Eur. J. Cancer.

[2] Center M.M., Jemal A., Lortet-Tieulent J., Ward E., Ferlay J., Brawley O., Bray F. (2012). International variation in prostate cancer incidence and mortality rates. Eur. Urol..

[3] Zhou C.K., Check D.P., Lortet-Tieulent J., Laversanne M., Jemal A., Ferlay J., Bray F., Cook M.B., Devesa S.S. (2016). Prostate cancer incidence in 43 populations worldwide: An analysis of time trends overall and by age group. Int. J. Cancer.

[4] Davidson P., Gabbay J. (2004). Should Mass Screening for Prostate Cancer be Introduced at the National Level?.

[5] Gondos A., Krilaviciute A., Smailyte G., Ulys A., Brenner H. (2015). Cancer surveillance using registry data: Results and recommendations for the Lithuanian national prostate cancer early detection programme. Eur. J. Cancer.

[6] Fund, N.H.I. Report of Early Prostate Cancer Detection Programme. http://www.vlk.lt/veikla/veiklos-sritys/prevencines-programos/priesines-liaukos-vezio-ankstyvosios-diiagnostikos-programa/Documents/0319%20pries%20liaukatask18.pdf.

[7] Patasius A., Innos K., Barchuk A., Ryzhov A., Leja M., Misins J., Yaumenenka A., Smailyte G. (2019). Prostate cancer incidence and mortality in the Baltic states, Belarus, the Russian Federation and Ukraine. BMJ Open.

[8] Doll R., Cook P. (1967). Summarizing indices for comparison of cancer incidence data. Int. J. Cancer.

[9] Kim H.J., Fay M.P., Feuer E.J., Midthune D.N. (2000). Permutation tests for joinpoint regression with applications to cancer rates. Stat. Med..

[10] Brawley O.W. (1997). Prostate carcinoma incidence and patient mortality: The effects of screening and early detection. Cancer.

[11] Collins M.M., Barry M.J. (1996). Controversies in prostate cancer screening. Analogies to the early lung cancer screening debate. JAMA.

[12] Gann P.H. (1997). Interpreting recent trends in prostate cancer incidence and mortality. Epidemiology.

[13] Schroder F.H., Hugosson J., Roobol M.J., Tammela T.L., Zappa M., Nelen V., Kwiatkowski M., Lujan M., Maattanen L., Lilja H. (2014). Screening and prostate cancer mortality: Results of the European Randomised Study of Screening for Prostate Cancer (ERSPC) at 13 years of follow-up. Lancet.

[14] Ilic D., Djulbegovic M., Jung J.H., Hwang E.C., Zhou Q., Cleves A., Agoritsas T., Dahm P. (2018). Prostate cancer screening with prostate-specific antigen (PSA) test: A systematic review and meta-analysis. BMJ.

[15] Welch H.G., Albertsen P.C. (2009). Prostate cancer diagnosis and treatment after the introduction of prostate-specific antigen screening: 1986-2005. J. Natl. Cancer Inst..

[16] Mettlin C. (2000). Impact of screening on prostate cancer rates and trends. Microsc. Res. Tech..

[17] Farkas A., Schneider D., Perrotti M., Cummings K.B., Ward W.S. (1998). National trends in the epidemiology of prostate cancer, 1973 to 1994: Evidence for the effectiveness of prostate-specific antigen screening. Urology.

[18] Djavan B. (2011). Screening for prostate cancer: Practical analysis of the ERSPC [corrected] and PLCO trials. Eur. Urol..

[19] Steele R.J., McClements P.L., Libby G., Black R., Morton C., Birrell J., Mowat N.A., Wilson J.A., Kenicer M., Carey F.A. (2009). Results from the first three rounds of the Scottish demonstration pilot of FOBT screening for colorectal cancer. Gut.

